# Medication process-related burden among informal caregivers of people with dementia: a nationwide cross-sectional survey in Germany

**DOI:** 10.1186/s12913-026-14325-4

**Published:** 2026-03-14

**Authors:** Tim Kossmann, Ann-Kathrin Zenk, Thilo Bertsche

**Affiliations:** 1 Drug Safety Center, Medical Faculty, University Hospital Leipzig, Leipzig University, Brüderstraße 32, 04103 Leipzig, Germany; 2 Clinical Pharmacy Department, Institute of Pharmacy, Medical Faculty, Leipzig University, Brüderstraße 32, 04103 Leipzig, Germany

**Keywords:** Caregiver burden, Medication process-related burden, Dementia, Medication, Community nurses, Community pharmacies

## Abstract

**Background:**

The medication process (MP) comprises all patient-related activities involved in medication use, from obtaining and organising medications to their correct administration and monitoring. In dementia, these responsibilities are frequently transferred to informal caregivers, adding to their already considerable burden from demanding care tasks. Although general caregiver burden is well documented and commonly assessed, little is known about the specific burden associated with MP-related tasks.

**Methods:**

Informal caregivers of people with dementia were asked to participate in a nationwide, anonymous, self-assessment survey. Here, we aimed to (i) identify key contributors to MP-related burden and correlate MP-related burden with the general caregiver burden, (ii) assess whether MP-related burden is different between informal caregivers who receive help from community nurses and those who do not and (iii) examine which community pharmacy services are deemed most valuable by informal caregivers of people with dementia.

**Results:**

In total, 180 informal caregivers, aged 67.6 ± 11.1 years and predominantly female (76.7%), participated. (i) In the MP, 27.3% of informal caregivers perceived communicating medication-related information to the patient as a task with (very) high burden. Obtaining prescribed medication from the pharmacy and preparing medication doses for intake were perceived as (very) high burden for 4.4% and 5.7% of informal caregivers, respectively. There was no correlation between MP-related burden and general caregiver burden (*r* = 0.157; *p* = 0.053). (ii) There was no difference in perceived MP-related burden between informal caregivers who received support from community nurses and those who did not (H (5) = 1,760; *p* = 0.881). (iii) When visiting community pharmacies, 73.5% and 70.6% of informal caregivers deemed “fast service” and “comprehensive counselling for prescription medicines” to be (very) important.

**Conclusion:**

With respect to MP-related burden, patient-related tasks were perceived as more burdensome than organisational tasks. Support from community nurses did not affect the perceived MP-related burden. Community pharmacies, through low-threshold counselling and structured collaboration with prescribers, may play a key role in alleviating MP-related burden.

**Registry:**

German Register of Clinical Trials (DRKS.de), DRKS00036251, Registration date: 09 July 2025.

**Supplementary Information:**

The online version contains supplementary material available at 10.1186/s12913-026-14325-4.

## Background

The medication process (MP) comprises all patient-related activities involved in medication use, from obtaining and organising medications to their correct administration and monitoring. This process can represent a considerable burden for patients [1], particularly when the medication regimen becomes more complex. In people with dementia, these responsibilities are often transferred to informal caregivers such as relatives, who, by taking on the practical tasks of the medication process, place themselves at risk of experiencing MP-related burden [2]. 

It is well established that informal caregivers of people with dementia experience considerable burden due to the physically and emotionally demanding aspects of nursing care [3]. This general caregiver burden has been shown to be associated with adverse outcomes for informal caregivers [4]. While previous studies have examined caregiver burden in relation to assistance with patients’ medication intake, few studies have addressed the specific MP-related burden arising from tasks within the medication process in dementia care and its contribution to overall caregiver burden [5]. 

Furthermore, while the impact of community nurses on caregiver burden has been investigated in interventional trial settings [6], data on informal caregivers’ perspectives regarding their impact on MP-related burden in real-world settings remain scarce. Similarly, although small pharmacist-led interventions have demonstrated benefits in reducing general caregiver burden in clinical trial settings [7], little is known about which routine service tasks from community pharmacies informal caregivers supporting people with dementia value the most.

The purpose of this study was to.

(i) explore which components of the MP contribute most to the perceived MP-related burden of informal caregivers and compare the MP-related burden to the general caregiver burden;
(ii) assess how many informal caregivers received support from community nurses and compare both the general caregiver burden and the MP-related burden experienced by informal caregivers who received support with those who did not;
(iii) examine which community pharmacy services are deemed most valuable by informal caregivers of people with dementia,

with the greater goal of collecting information for the future development of interventions for informal caregivers with the aim to improve the MP for people with dementia.

## Methods

### Study design

A prospective, nationwide, cross-sectional survey study was performed in Germany.

### Participants and setting

Informal caregivers of people with dementia were invited to complete an anonymous questionnaire, either digitally or in hard copies. Self-help groups nationwide were contacted to distribute the questionnaire to their members. Hard copies were returned by post to the primary investigators. A list of all self-help groups was accessed via the German society of Alzheimer’s disease (Deutsche Alzheimer Gesellschaft e.V.). As effect sizes for this approach were unknown and the study was exploratory, no formal sample size calculation was undertaken.

The survey was conducted anonymously. The first page of the questionnaire provided detailed study information regarding objectives, voluntary participation, and data protection. Completion of the questionnaire was considered as implied informed consent. The questionnaire collected informal caregiver-reported information on caregiving experiences and medication-related processes only; no identifiable or personal data of people with dementia were obtained.

### Eligibility criteria

We contacted support groups for informal caregivers of people with dementia. We asked the support groups to distribute the questionnaire to all informal caregivers over the age of 18 who support people with dementia with day-to-day tasks, especially those related to medication. We made no a priori selection regarding the aetiology of the dementia illness.

### Questionnaire

The questionnaire used in this study was newly developed by the authors in collaboration with community pharmacists and a local self-help group for informal caregivers of people with dementia.

For medication process (MP)-related burden and community pharmacy services, items were generated based on experiences from pharmaceutical practice by the authors and four additional pharmacists. To ensure relevance for the target population, these items were reviewed with a social worker leading a caregiver support group. The questionnaire was subsequently pre-tested for scientific accuracy with four pharmacists and for comprehensibility with two informal caregivers of people with dementia, resulting in minor wording adjustments. In addition to the self-developed items, the validated short-form Zarit Burden Interview was incorporated to assess general caregiver burden [8]. 

Medication process (MP)-related burden (i) was evaluated using eight items addressing MP-related tasks, rated on five-point Likert scales ranging from 1 (no burden) to 5 (very high burden). For comparison with general caregiver burden, individual item scores were summed, with higher values indicating greater MP-related burden.

Support from community nurses (ii) was assessed by a question on the frequency of supportive visits. Informal caregiver could select whether they received support once weekly, once-, twice-daily, three times daily, in another interval or no support at all. Supporting tasks comprised direct nursing care (health assessment, symptom monitoring, medication preparation, and assistance with activities of daily living) as well as practical support, including obtaining medications from community pharmacies. The impact of community nurse support was evaluated by comparing general caregiver burden and MP-related burden between informal caregiver who received any extend of support and those who did not.

Informal caregivers’ perceived importance of community pharmacy services (iii) was assessed by asking informal caregiver to rate various pharmacy services on a Likert scale ranging from 1 (very important) to 5 (not important at all).

For the purposes of this study, community pharmacy services refer to professional pharmaceutical care activities offered in publicly accessible pharmacies beyond medication dispensing. These services particularly include medication counselling and organisational support services, such as mobile ordering and home delivery, that are relevant to informal caregivers of people with dementia. The complete translated questionnaire is provided in Supplementary Appendix 1.

### Statistical analysis

Calculations were performed via SPSS (Statistical Package for the Social Science, Version 29.0.2.0; IBM, Armonk, New York, USA). Normally distributed values (Informal caregiver age, people with dementia age and caregiver burden from ZBI-7) are reported as the means with their standard deviation, not normally distributes values as median with the 25th and 75th percentiles (Q25/Q75). When comparing general caregiver burden and MP-related burden, Pearson’s correlation was applied. The Mann‒Whitney U test was applied to compare the general caregiver burden and MP-related burden between informal caregivers receiving support from community nurses and those who did not. A p-value ≤ 0.05 was considered to indicate significance.

## Results

### Recruitment

**Fig. 1:**
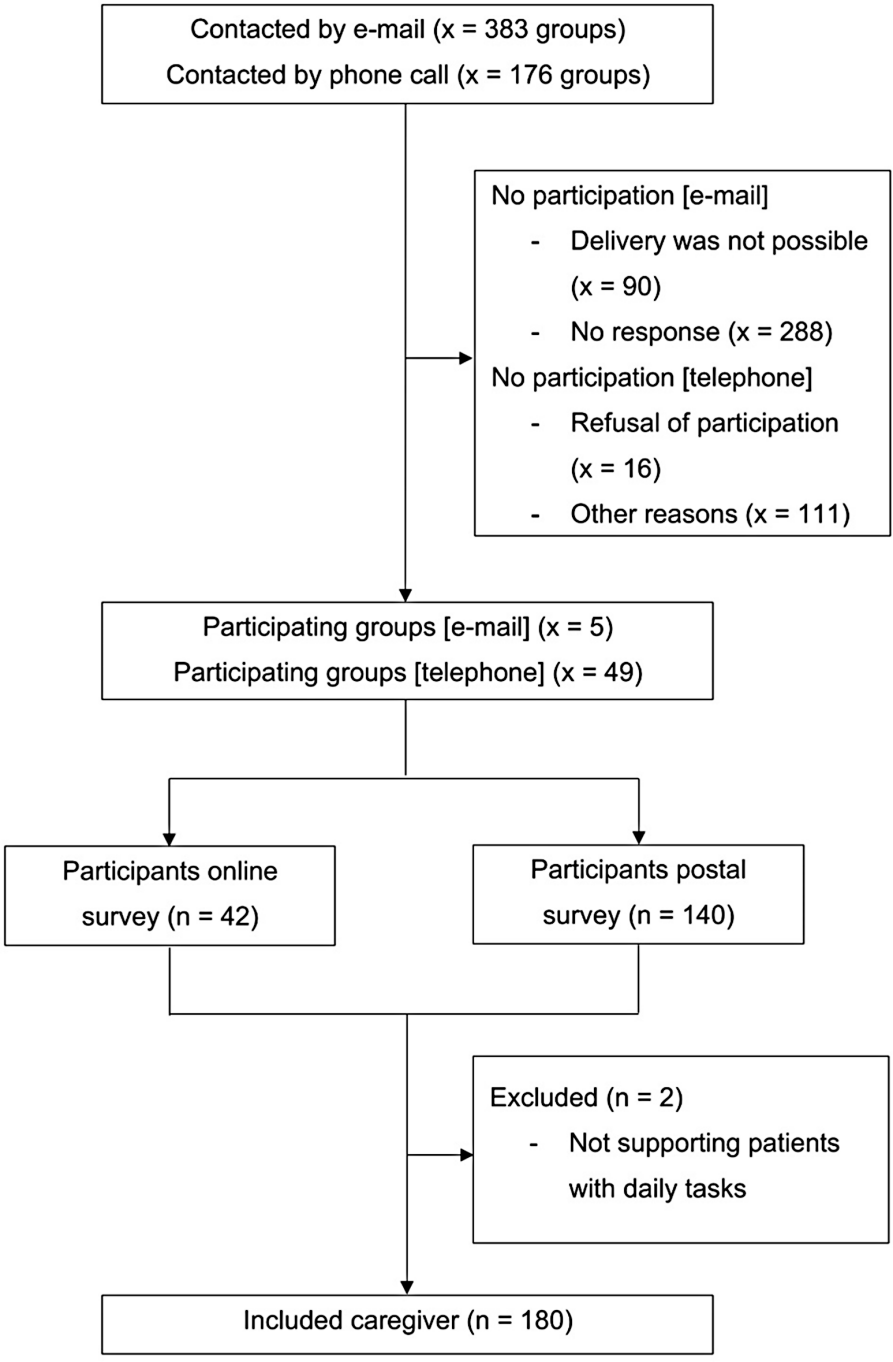
Recruitment of the study population

We contacted 383 support groups by e-mail. Where a telephone number was available, we also contacted the group by telephone. In total, fifty-four support groups for informal caregivers of people with dementia agreed to participate with their members, which accounted for 14% of total groups listed on the website of the German Society of Alzheimer’s e.V. This resulted in a total of 180 informal caregivers completing our questionnaire anonymously (Fig. 1).

### Informal caregiver and patient characteristics

Informal caregivers from 14 out of 16 federal states of Germany participated in this study. Informal caregivers and patients baseline characteristics are listed in Table 1.

**Table 1:** Characteristics of informal caregivers and people with dementia

**Characteristics of people with dementia**	***n*** ** = 180**
**Age**	
Mean ± SD	80.0 ± 8.1
**Sex**	
Female (%)	78 (43.3)
Male (%)	101 (56.1)
Not specified (%)	1 (0.6)
**Medications per patient**	
Median (Q25/Q75)	5 (4/8)
**Characteristics of informal caregivers**	*n* = 180
**Age**	
Mean ± SD	67.6 ± 11.1
**Sex**	
Female (%)	138 (76.7)
Male (%)	39 (21.7)
Not specified (%)	3 (1.7)
**Caregiver burden**	
Zarit-Burden Interview mean ± SD	14.6 ± 5.1
**Relationship to the patient**	
Spouse (%)	114 (63.3)
Child/Grandchild (%)	55 (30.6)
Friend/Neighbour (%)	4 (2.2)
Brother/Sister (%)	2 (1.1)
Parent (%)	0 (0.0)
Not specified (%)	5 (2.8)
**Highest education level**	
Vocational training (%)	61 (33.9)
University degree (%)	50 (27.8)
Secondary school diploma (%)	41 (22.8)
University entrance qualification (%)	23 (12.8)
No education certificate (%)	4 (2.2)
Not specified (%)	1 (0.6)
**Employment status**	
Retired (%)	110 (61.1)
Part-time employment (%)	33 (18.3)
Full-time employment (%)	20 (11.1)
Currently unemployed (%)	15 (8.3)
In education/training (%)	0 (0.0)
Not specified (%)	2 (1.1)
**Medications per informal caregiver**	
Median (Q25/Q75)	2 (1/4)

### Components of the MP and their contribution to burden

**Fig. 2:**
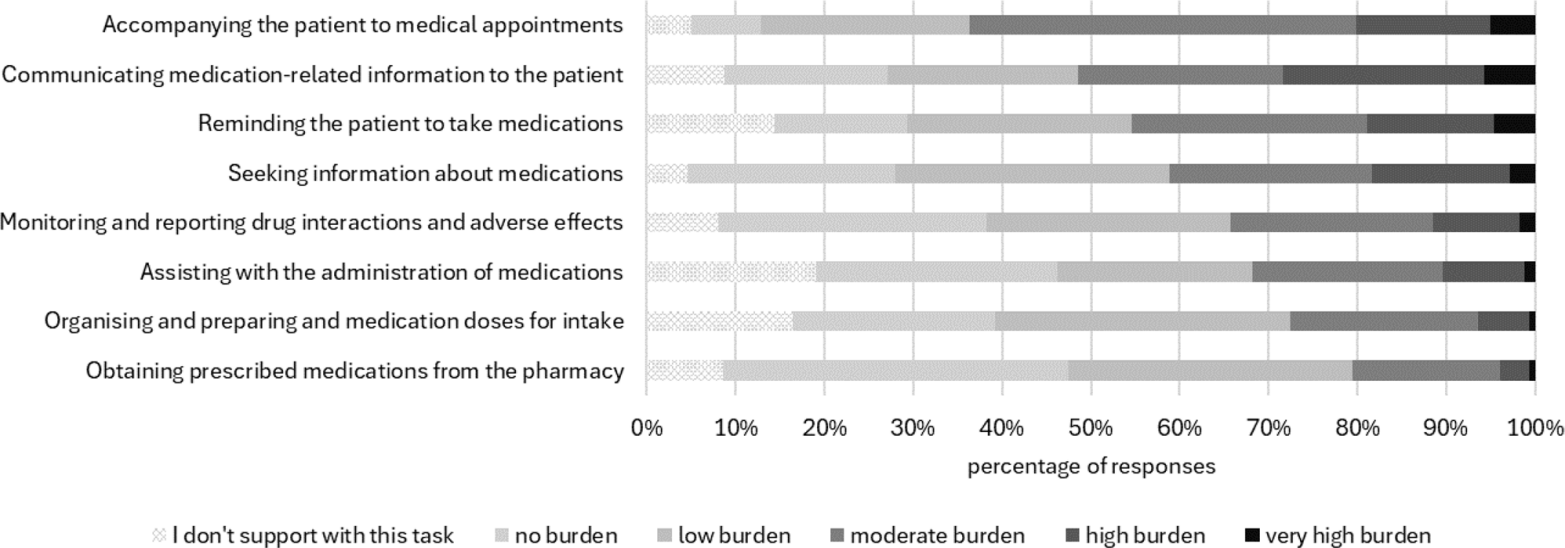
Components of the medication process (MP) and their contribution to burden

Among the tasks in the MP, communicating medication-related information to the patient was perceived as very high or high burden by 27.3% of the informal caregivers. Furthermore, accompanying the patient to medical appointments and reminding the patient to take the medication, were perceived as similarly burdensome (very high or high burden in 20.0% and 18.3% of informal caregivers, respectively). Obtaining prescribed medications from the pharmacy or organising and preparing medication doses for intake were perceived as very high or high burden in 4.4% and 5.7% of informal caregivers, respectively (Fig. 2).

There was no statistically significant correlation between the general caregiver burden and the MP-related burden (*r* = 0.157; *p* = 0.053).

### Influence of community nurses on burden

In our study, 37.8% of informal caregivers received help from community nurses in the MP of people with dementia. Informal caregivers and people with dementia received help most commonly once or twice daily (9.4% and 9.9%) (Table 2).

**Table 2:** Community nurse support

Frequency of visits from community nurses	Percentage of caregivers
No support from community nurses	62.2%
Once weekly	6.8%
Once daily	9.4%
Twice daily	9.9%
Three times daily	5.7%
Other Interval	6.0%

Support from community nurses was assessed by asking caregivers about the extend of support they receive from community nurses in the medication process of the people with dementia

There was no statistically significant difference in the informal caregivers perceived general caregiver burden (U = 3837.5, *p* = 0.066) or MP-related burden (U = 2896.0, *p* = 0.595) between informal caregivers who received help from community nurses and those who did not.

### Informal caregivers’ ratings of the importance of community pharmacy services

When asked about the importance of different services of a community pharmacy, 73.5% of informal caregivers deemed it very important or important to receive fast service. Furthermore, detailed consultations related to prescription drugs (70.6%) and OTC medications (62.6%) were valued as (very) important by informal caregivers. Services offering organisational support, such as apps, mail or phone-based ordering or home delivery, were deemed (very) important by 40.8% and 39.5%, respectively.

## Discussion

Our study cohort revealed high levels of general caregiver burden and burden arising from the MP. With respect to MP-related burden, tasks that require the informal caregiver to have patient interactions were perceived as more burdensome than organisational tasks. Furthermore, support from community nurses did not significantly reduce the MP-related burden, and informal caregivers found fast service and extensive counselling to be more important than organisational services in pharmacies.

### Components of the MP and their contribution to burden

In our study, the most burdensome components of medication management were tasks that required direct interaction with the person with dementia, including explaining medication-related information, accompanying them to physician visits, and reminding them to take their medications. In contrast, organisational tasks such as collecting prescriptions or preparing medication were rarely perceived as highly burdensome. This suggests that interpersonal and decision-related aspects of medication management impose a greater contribution to MP-related burden than organisational responsibilities alone. These findings are consistent with the broader caregiver burden literature, in which patient-related and interpersonal demands are described as particularly challenging [8]. 

One explanation may be that informal caregivers are frequently required to make health-related decisions on behalf of the person with dementia and subsequently communicate and justify these decisions. Within the MP, such responsibility may be especially distressing, as treatment decisions often have significant implications for disease progression, adverse effects, and quality of life. This burden appears to be amplified in high-pressure situations such as physician visits, where complex medical information must be processed and important decisions are often made within limited timeframes [9]. Interventions aimed at improving structured communication during clinical encounters, providing clear medication plans, and strengthening decision support for informal caregivers may therefore help to reduce MP- related burden.

Consistent with previous studies, MP-related burden showed only a weak association with overall caregiver burden, suggesting that medication management represents a partially independent dimension of caregiving stress [8, 10]. Our findings highlight the MP as a distinct and clinically relevant target for supportive interventions, which may be guided by a dedicated MP-related burden score to identify specific stressors and tailor targeted support strategies [12]. 

### Impact of community nurse services on MP-related burden

Our study revealed no difference in either general caregiver burden or MP-related burden between informal caregivers who received community nurse support and those who did not, consistent with previous findings [11, 12]. The difference in general caregiver burden narrowly missed statistical significance, which may indicate a trend that could be attributable to the limited sample size. This may be explained by the scope of community nursing services: while nurses commonly assist with personal care and organisational aspects of daily management, potentially contributing to reductions in overall caregiver burden, the most burdensome MP tasks, particularly communication of medication-related information and accompanying people with dementia to physician visits, remain primarily with informal caregivers. Consequently, although formal nursing support is essential for physically demanding aspects of care, it may not directly address the interpersonal and decision-related components that drive MP-related burden. Expanding the involvement of well-qualified nurses in medication-related communication and decision-making processes may therefore represent an important strategy to reduce MP-related caregiver burden.

### Informal caregivers’ ratings of the importance of community pharmacy services

Owing to their low threshold accessibility, community pharmacies are a central contact point in the health care system for informal caregivers [13]. Owing to their central role in the MP, they hold particular potential to alleviate MP-related caregiver burden.

In accordance with previous studies, time pressure has emerged as a central problem for informal caregivers in community pharmacies since the constant supervision required by people with dementia leaves informal caregivers with little opportunity to manage daily tasks [14, 15]. 

While studies in nursing settings have demonstrated relief when informal caregivers can temporarily leave people with dementia under professional supervision at home [16], pharmacies may offer a complementary approach. Encouraging informal caregivers to bring patients along and actively involving them in consultations could alleviate the stress of leaving patients unattended. At the same time, it helps prevent the patients’ exclusion from MP-related decisions, thereby supporting their sense of independence, a loss of which many people with dementia fear [17]. Such approaches, however, need to be adapted carefully to the stage of dementia, as active participation may not be feasible in more advanced disease.

In addition, pharmacies could facilitate structured communication with prescribers, for example, regarding adverse drug reactions or other drug-related problems [18]. Established and reimbursed services, such as medication reviews in patients with polypharmacy, provide a framework for such systematic collaboration, which would further relieve informal caregivers of part of their responsibility. Previous findings have highlighted the positive effects of pharmacist-based interventions on caregiver burden and urge a stronger implementation of pharmacies in the MP [7, 19], underscoring the possibility that pharmacies can play a meaningful role in alleviating MP-related and general caregiver burden.

Overall, our findings suggest that community pharmacies may contribute most effectively in the MP by prioritizing services directly relevant to informal caregivers, such as timely access and comprehensive counselling, while also creating opportunities for patient involvement where appropriate.

### Limitations

The main limitation of this study is that participants were recruited exclusively from informal caregiver self-help groups, thereby capturing a population already actively engaged in support networks. Informal caregivers who do not participate in such groups, due to lack of interest, access, or awareness, may experience different patterns of burden and healthcare engagement. In addition, the distribution of questionnaires within the groups was not standardized, raising the possibility of selective distribution by group leaders. Finally, although the number of participating support groups was sufficient to provide an overview of this population, the overall sample of 180 informal caregivers remains relatively small in relation to the total population of dementia caregivers in Germany, which may limit generalizability.

## Conclusion

MP-related burden appears to constitute a partly independent dimension of caregiver burden, primarily driven by communicational- and informational- rather than organisational tasks. While community nurse services do not address these aspects specifically and therefore fails to alleviate caregiver-perceived MP-related burden, community pharmacies have the potential to offer targeted support through accessible counselling, structured collaboration with prescribers, and the inclusion of people with dementia in the MP.

## Supplementary Information

Below is the link to the electronic supplementary material.

Supplementary Material 1

## Data Availability

The datasets used and/or analysed during the current study are available from the corresponding author on reasonable request.

## Abbreviations

MP: medication process

## References

[1] Gebreyohannes EA, Gebresillassie BM, Mulugeta F, Dessu E, Abebe TB. Treatment burden and health-related quality of life of patients with multimorbidity: a cross-sectional study. Qual Life Res. 2023;32:3269–77. 10.1007/s11136-023-03473-3. 37405663 10.1007/s11136-023-03473-3 PMC10522511

[2] Gillespie R, Mullan J, Harrison L. Managing medications: the role of informal caregivers of older adults and people living with dementia. A review of the literature. J Clin Nurs. 2014;23:3296–308. 10.1111/jocn.12519. 24354583 10.1111/jocn.12519

[3] Adelman RD, Tmanova LL, Delgado D, Dion S, Lachs MS. Caregiver burden: a clinical review. JAMA. 2014;311:1052–60. 10.1001/jama.2014.304. 24618967 10.1001/jama.2014.304

[4] Schulz R, Beach SR. Caregiving as a risk factor for mortality: the Caregiver Health Effects Study. JAMA. 1999;282:2215–9. 10.1001/jama.282.23.2215. 10605972 10.1001/jama.282.23.2215

[5] Onda M, Inoue M, Zouchi K, Shoji M, Maeda H. Understanding the burden and influencing factors in family caregivers’ medication assistance for patients with dementia: a survey study. BMC Geriatr. 2024;24:975. 10.1186/s12877-024-05570-5. 39609762 10.1186/s12877-024-05570-5 PMC11603863

[6] Kazemi A, Azimian J, Mafi M, Allen K-A, Motalebi SA. Caregiver burden and coping strategies in caregivers of older patients with stroke. BMC Psychol. 2021;9:51. 10.1186/s40359-021-00556-z. 33794995 10.1186/s40359-021-00556-z PMC8017750

[7] Nanaumi Y, Yoshitani A, Onda M. Impact of interventions by a community pharmacist on care burden for people with dementia: development and randomized feasibility trial of an intervention protocol. Pilot Feasibility Stud. 2022;8:118. 10.1186/s40814-022-01071-7. 35655244 10.1186/s40814-022-01071-7 PMC9161485

[8] Chiao C-Y, Wu H-S, Hsiao C-Y. Caregiver burden for informal caregivers of patients with dementia: A systematic review. Int Nurs Rev. 2015;62:340–50. 10.1111/inr.12194. 26058542 10.1111/inr.12194

[9] Look KA, Stone JA. Medication management activities performed by informal caregivers of older adults. Res Social Adm Pharm. 2018;14:418–26. 10.1016/j.sapharm.2017.05.005. 28528023 10.1016/j.sapharm.2017.05.005 PMC5690891

[10] Steinsheim G, Malmedal W, Follestad T, Olsen B, Saga S. Factors associated with subjective burden among informal caregivers of home-dwelling people with dementia: a cross-sectional study. BMC Geriatr. 2023;23:644. 10.1186/s12877-023-04358-3. 37817101 10.1186/s12877-023-04358-3 PMC10565959

[11] Robinson KM, Buckwalter KC, Reed D. Predictors of use of services among dementia caregivers. West J Nurs Res. 2005;27. 10.1177/0193945904272453. 10.1177/0193945904272453 15695566

[12] Karrer L, Dietzel N, Wolff F, Kratzer A, Hess M, Gräßel E, Kolominsky-Rabas P. Wenn es nicht mehr alleine geht – Inanspruchnahme ambulanter Unterstützungsangebote von Menschen mit Demenz: der Bayerische Demenz Survey (BayDem). [Use of Outpatient Care Services by People with Dementia: Results of the Bavarian Dementia Survey (BayDem)]. Gesundheitswesen. 2020;82:40–9. 10.1055/a-1071-7851. 31863444 10.1055/a-1071-7851

[13] Eggert S, Sulmann D, Teubner C. Medikation in der häuslichen Pflege aus Sicht pflegender Angehöriger. Monit Pflege 07. 2019;5(10):24–31.

[14] Lindeza P, Rodrigues M, Costa J, Guerreiro M, Rosa MM. Impact of dementia on informal care: a systematic review of family caregivers’ perceptions. BMJ Supportive Palliat Care. 2020;14:e38–49. 10.1136/bmjspcare-2020-002242. 10.1136/bmjspcare-2020-002242 33055092

[15] Smith ML, Southerland JL, Neelamegam M, Han G, Lee S, Kew CL, et al. Caregiving burdens of task time and task difficulty among paid and unpaid caregivers of persons living with dementia. Front Public Health. 2025;13:1615187. 10.3389/fpubh.2025.1615187. 40703190 10.3389/fpubh.2025.1615187 PMC12283594

[16] Vaismoradi M, Logan PA. Contributions of Specialized Nurses to Medication Management for Older People in Home Care: A Mixed-Method Systematic Review. Risk Manage Healthc Policy. 2025;18:445–70. 10.2147/RMHP.S508170. 10.2147/RMHP.S508170 PMC11831921 39963544

[17] Maxfield M, Peckham A, James DL. Terror Is a Better Word: A Qualitative Analysis of Dementia-Related Anxiety. Gerontologist. 2024;64:gnad146. 10.1093/geront/gnad146. 37878745 10.1093/geront/gnad146 PMC11020257

[18] Smith F, Francis S-A, Gray N, Denham M, Graffy J. A multi-centre survey among informal carers who manage medication for older care recipients: problems experienced and development of services. Health Soc Care Commun. 2003;11:138–45. 10.1046/j.1365-2524.2003.00415.x. 10.1046/j.1365-2524.2003.00415.x 14629216

[19] Kiiski A, Luoma E, Airaksinen M, Pohjanoksa-Mäntylä M, Desselle S, Kivelä S-L. Caregivers’ challenges in engaging with the health system to optimise medication management of older care recipients: a qualitative study including home visits. BMJ Open. 2025;15:e093122. 10.1136/bmjopen-2024-093122. 40578872 10.1136/bmjopen-2024-093122 PMC12207135

