# Supplementary material for: Medication process-related burden among informal caregivers of people with dementia: a nationwide cross-sectional survey in Germany
Source: BMC Health Serv Res. 2026 Mar 14;26:487. doi: 10.1186/s12913-026-14325-4 (PMC13063673; doi:10.1186/s12913-026-14325-4)
Supplement: Supplementary file 1 — Supplementary Material 1 [file 12913_2026_14325_MOESM1_ESM.pdf]

General Information

1 Do you help a relative who suffers from dementia with everyday tasks?
- ❑ Yes
- ❑ No

If you answer 'No' to this question, you do not need to complete the rest of the questionnaire!

Below, we ask a few questions about you.

2 How old are you? ___________

3 In which federal state do you life?

- ❑ Baden-Württemberg
- ❑ Bavaria
- ❑ Berlin
- ❑ Brandenburg
- ❑ Bremen
- ❑ Hamburg
- ❑ Hesse
- ❑ Mecklenburg-Vorpommern
- ❑ Lower-Saxony
- ❑ North Rhine-Westphalia
- ❑ Rhineland-Palatinate
- ❑ Saarland
- ❑ Saxony
- ❑ Saxony-Anhalt
- ❑ Schleswig-Holstein
- ❑ Thuringa

4 What is your gender?
- ❑ male
- ❑ female
- ❑ divers

5 What is your relationship to the relative you are supporting?
The relative is your ...
- ❑ Spouse
- ❑ Parent
- ❑ Brother / Sister
- ❑ Child
- ❑ Friend or Neighbour
- ❑ Other: _________________

6 What is your highest level of education?
- ❑ No education certificate
- ❑ Secondary school diploma
- ❑ University entrace qualification
- ❑ Vocational training
- ❑ University degree

7 What is your current employment status?
- ❑ Full-time employment
- ❑ Part-time employment
- ❑ Retired
- ❑ In Training
- ❑ Currently unemployed

8 *How many medications you take on a regular basis?*
Please tick the appropriate box:

0	1	2	3	4	5	6	7	8	9	10	11	12	13	14	15	16	17	18	19	20

Patient (person with dementia)

Below, we would like to ask you a few questions about your relative who has dementia.

1 How old is your relative? ___________

2
- ❑ What is your relative's gender?
- ❑ male
- ❑ female
- ❑ divers

4	Which type of dementia does your relative suffer from?<br>❑ I don't know<br>❑ from the Alzheimer's spectrum<br>❑ vasculare dementia<br>❑ Frontotemporal dementia (FTD)<br>❑ Lewy-Body dementia<br>❑ Mild Cognitive Impairment (MCI)<br>❑ Mixed form
5	*How would you personally rate the severity of your relative's dementia on a scale of 1-5?*<br>*1 = no impairment due to dementia*<br>*5 = most severe impairment due to dementia*<br>*Please tick the appropriate box:*<br>❑ 1 ❑ 2 ❑ 3 ❑ 4 ❑ 5
6	*How many medications does your relative take on a regular basis?*<br>*Please tick the appropriate box:*<br>0 | 1 | 2 | 3 | 4 | 5 | 6 | 7 | 8 | 9 | 10 | 11 | 12 | 13 | 14 | 15 | 16 | 17 | 18 | 19 | 20

Medication knowledge and Medication process related burden

The following section deals with your knowledge of your relative's medication and possible burdens for you.

1 Do you receive support from a community nurse when caring for your relative?

- ❑ No
- ❑ Once a day
- ❑ Twice a day
- ❑ 3 times a day
- ❑ 1 time a week
- ❑ Other: ________________________

2 *How burdensome are the following support activities for you?*
Please tick the appropriate box:

	I do not assist with this activity.	No burden	Low burden	Moderate burden	High burden	Very high burden
1. Accompanying the patient to medical appointments	❑	❑	❑	❑	❑	❑
2. Obtaining prescribed medications from the pharmacy	❑	❑	❑	❑	❑	❑
3. Preparing medications for administration	❑	❑	❑	❑	❑	❑
4. Reminding the patient to take medications	❑	❑	❑	❑	❑	❑
5. Monitoring and reporting potential drug interactions and side effects	❑	❑	❑	❑	❑	❑
6. Seeking information about medications (eg. adverse reactions)	❑	❑	❑	❑	❑	❑
7. Communicating medication-related information to the patient	❑	❑	❑	❑	❑	❑
8. Assisting with the administration of medications	❑	❑	❑	❑	❑	❑

3 How well do you know the purpose of each of your relative's medications?
I know the purpose...

- ❑ ... of every medicine
- ❑ ... of most medicines
- ❑ ... of about half of all medicines
- ❑ ... of a few medicines
- ❑ ... of no medicines

4	How well informed do you feel about the side effects of your relative's medication?<br>❑ Very good<br>❑ Good<br>❑ Partly<br>❑ Poor<br>❑ Very poor
5	Do you know the names of the medications your relative is taking?<br>I know the name...<br>❑ ... of every medicine<br>❑ ... of most medicines<br>❑ ... of about half of all medicines<br>❑ ... of a few medicines<br>❑ ... of no medicines
6	Where do you obtain information about your relative's medication?<br>❑ I do not seek information<br>❑ From my doctor<br>❑ From my pharmacist<br>❑ From the internet<br>❑ From package inserts/patient information leaflets<br>❑ Other: _____________

Taking your own medication	
1	How well do you adhere to taking your doctor-prescribed medication regularly?<br>❑ I never forget to take my medication<br>❑ I forget to take my medication once a month<br>❑ I forget to take my medication 2-4 times a month<br>❑ I forget to take my medication once a week<br>❑ I forget to take my medication several times a week
Self-medication refers to medicines you take without a doctor's prescription.<br>This also includes nutrients, vitamins, medicinal teas and herbal products from pharmacies, drugstores, supermarkets or the internet.	
2	How often have you self-medicated in the last 2 weeks?<br>❑ No self-medication used<br>❑ Less than once a week<br>❑ Approximately once a week<br>❑ Approximately 3 times a week<br>❑ Daily<br>❑ Several times a day
3	Please rate the following statement.<br>'Self-medication helps me to better cope with my everyday life and caring for my relative.'<br>❑ I completely agree<br>❑ I agree<br>❑ Neutral<br>❑ I disagree<br>❑ I completely disagree
4	Do you take any medications or supplements to prevent dementia?<br>❑ Yes<br>❑ No
5	If so, what medications are you taking? (Multiple answers possible)B-Vitamine<br>❑ Vitamin D<br>❑ Other vitamins<br>❑ Ginkgo<br>❑ Minerals (magnesium, calcium, selenium, zinc, etc.)<br>❑ Medicinal substances from traditional Chinese medicine<br>❑ Homeopathic medicines<br>❑ Other:
6	How important are the following when visiting a community pharmacy?<br>*Please tick the appropriate box:*

	Very important	Important	Partly/partly	not important	Not important at all	Not specified
1. Fast Service	❑	❑	❑	❑	❑	❑
2. Detailed consultation on self-medication	❑	❑	❑	❑	❑	❑
3. Detailed consultation on prescription-medication	❑	❑	❑	❑	❑	❑
4. Detailed consultation on non-medicinal treatment options	❑	❑	❑	❑	❑	❑
5. Home delivery service	❑	❑	❑	❑	❑	❑
6. Ordering medicines via email, telephone or apps	❑	❑	❑	❑	❑	❑

Zarit Burden Interview	
1	Do you feel you don't have enough time for yourself?<br>❑ Never<br>❑ Rarely<br>❑ Sometimes<br>❑ Frequently<br>❑ Nearly Always
2	Do you feel stressed between caring and meeting other responsibilities?<br>❑ Never<br>❑ Rarely<br>❑ Sometimes<br>❑ Frequently<br>❑ Nearly Always
3	Do you feel your relative affects your relationship with others in a negative way?<br>❑ Never<br>❑ Rarely<br>❑ Sometimes<br>❑ Frequently<br>❑ Nearly Always
4	Do you feel strained when you are around your relative?<br>❑ Never<br>❑ Rarely<br>❑ Sometimes<br>❑ Frequently<br>❑ Nearly Always
5	Do you feel your health has suffered because of your involvement with your relative?<br>❑ Rarely<br>❑ Sometimes<br>❑ Frequently<br>❑ Nearly Always
6	Do you feel you have lost control of your life since your relative's illness?<br>❑ Never<br>❑ Rarely<br>❑ Sometimes<br>❑ Frequently<br>❑ Nearly Always
7	Overall, how burdened do you feel in caring for your relative?<br>❑ Never<br>❑ Rarely<br>❑ Sometimes<br>❑ Frequently<br>❑ Nearly Always
